# Supplementary material for: Evolutionary changes in transcription factor coding sequence quantitatively alter sensory organ development and function
Source: eLife. 2017 Apr 13;6:e26402. doi: 10.7554/eLife.26402 (PMC5432213; doi:10.7554/eLife.26402)
Supplement: Supplementary file 1. — DOI: http://dx.doi.org/10.7554/eLife.26402.017 [file elife-26402-supp1.docx]

**Supplementary File 1.** **List of** **organisms for the tBLASTn search**

| **Organism** | **Taxonomic group** |
| --- | --- |
| *Nematostella vectensis* | Cnidaria |
| *Hydra magnipapillata* | Cnidaria |
| *Acropora digitifera* | Cnidaria |
| *Amphimedon queenslandica* | Porifera |
| *Sycon ciliatum* | Porifera |
| *Trichoplax adhaerens* | Placozoa |
| *Mnemiopsis leidyi* | Ctenophora |
| *Pleurobrachia bachei* | Ctenophora |
| *Lottia gigantea* | Mollusca |
| *Pinctada fucata* | Mollusca |
| *Aplysia californica* | Mollusca |
| *Capitella teleta* | Annelida |
| *Helobdella robusta* | Annelida |
| *Schmidtea mediterranea* | Platyhelminthes |
| *Schistosoma japonicum* | Platyhelminthes |
| *Clonorchis sinensis* | Platyhelminthes |
| *Schistosoma mansoni* | Platyhelminthes |
| *Caenorhabditis elegans* | Nematoda |
| *Trichinella spiralis* | Nematoda |
| *Tetranychus urticae* | Chelicerata |
| *Ixodes scapularis* | Chelicerata |
| *Daphnia pulex* | Crustacea |
| [*Acyrthosiphon pisum*](http://en.wikipedia.org/wiki/Acyrthosiphon_pisum) | Hemimetabola |
| *Rhodnius prolixus* | Hemimetabola |
| *Pediculus humanus* | Hemimetabola |
| *Tribolium castaneum* | Holometabola |
| *Apis mellifera* | Holometabola |
| *Nasonia vitripennis* | Holometabola |
| *Acromyrmex echinatior* | Holometabola |
| *Bombyx mori* | Holometabola |
| *Danaus plexippus* | Holometabola |
| *Heliconius melpomene* | Holometabola |
| *Glossina morsitans* | Diptera |
| *Aedes aegypti* | Diptera |
| *Anopheles gambiae* | Diptera |
| *Culex quinquefasciatus* | Diptera |
| *Drosophila grimshawi* | Drosophilinae |
| *Drosophila mojavensis* | Drosophilinae |
| *Drosophila virilis* | Drosophilinae |
| *Drosophila willstoni* | Drosophilinae |
| *Drosophila pseudoobscura* | Drosophilinae |
| *Drosophila persimilis* | Drosophilinae |
| *Drosophila ananassae* | Drosophilinae |
| *Drosophila erecta* | Drosophilinae |
| *Drosophila yakuba* | Drosophilinae |
| *Drosophila simulans* | Drosophilinae |
| *Drosophila sechellia* | Drosophilinae |
| *Homo sapiens* | Vertebrata |
| *Mus musculus* | Vertebrata |
| *Gallus gallus* | Vertebrata |
| *Xenopus laevis* | Vertebrata |
| *Xenopus tropicalis* | Vertebrata |
| *Danio rerio* | Vertebrata |
| *Petromyzon marinus* | Vertebrata |
| *Strongylocentrotus purpuratus* | Echinodermata |
| *Ciona intestinalis* | Tunicata |
| Branchiostoma floridae | Cephalochordata |
| *Saccoglossus kowalevskii* | Enteropneusta |
