# Supplementary material for: Evolutionary changes in transcription factor coding sequence quantitatively alter sensory organ development and function
Source: eLife. 2017 Apr 13;6:e26402. doi: 10.7554/eLife.26402 (PMC5432213; doi:10.7554/eLife.26402)
Supplement: Supplementary file 2. — DOI: http://dx.doi.org/10.7554/eLife.26402.018 [file elife-26402-supp2.docx]

**Supplementary File 2.** **Sources of DNA**

| **Gene** | **Organism** | **Sources** | **Rationale** |
| --- | --- | --- | --- |
| Ato | Drosophila melanogaster (fruit fly) | B. Hassan | reference |
| Amos | Drosophila melanogaster (fruit fly) | gDNA | Paralog /Ecdysozoan |
| Cato | Drosophila melanogaster (fruit fly) | gDNA | Paralog /Ecdysozoan |
| BmAto | Bombyx mori (silk moth) | F. Pignoni | most distant functional homolog |
| BfAth | Branchiostoma floridae (lancelet) | L. Holland | Cephalochoradata |
| MmAth1 | Mus musculus (mouse) | H. Zogbi | Vertebrates |
| MmAth5 | Mus musculus (mouse) | N. Brown | Vertebrates |
| HsAth1 | Homo sapiens (human) | H. Zogbi | Vertebrates |
| PdAth2 | Platynereis dumerilii (annelide worm) | M. Vervoort | Lophotrochozoa |
| AqbHLH1 | Amphimedon queenslandica (sponge) | B. Degnan | Porifera/"ancestral gene" |
| Tap | Drosophila melanogaster (fruit fly) | gDNA | Neurogenin family |
| Scute | Drosophila melanogaster (fruit fly) | Y. N. Jan | Achaete/Scute family |
