# Supplementary material for: Evolutionary changes in transcription factor coding sequence quantitatively alter sensory organ development and function
Source: eLife. 2017 Apr 13;6:e26402. doi: 10.7554/eLife.26402 (PMC5432213; doi:10.7554/eLife.26402)
Supplement: Supplementary file 3. — DOI: http://dx.doi.org/10.7554/eLife.26402.019 [file elife-26402-supp3.docx]

**Supplementary File 3.** **Antibodies used in this study**

| **Antibody** | **Concentration** | **Source** |
| --- | --- | --- |
| rat anti-ELAV | 1:100 | 7E8A10, DSHB, G. Rubin |
| mouse anti-Prospero | 1:4 | MR1A, DSHB, C. Doe |
| mouse anti-Seven-up | 1:500 | Y. Hiromi (Kanai et al., 2005) |
| guinea pig anti-Senseless | 1:1000 | H. Bellen (Nolo et al., 2000) |
| rabbit anti-MmAth1 | 1:200 | M. Hoshino (Yamada et al., 2014) |
| sheep anti-Ato | 1:250 | A. Jarman |
| rabbit anti-Amos | 1:1000 | A. Jarman (Lage et al., 2003) |
| sheep anti-Cato | 1:1000 | A. Jarman (zur Lage and Jarman, 2010) |
| rabbit anti-Rhodopsin 6 | 1:1000 | C. Desplan (Tahayato et al., 2003) |
| rabbit anti-HRP | 1:500 | 323005021 Jackson Immunoresearch |
| mouse anti-Repo | 1:5 | 8D12, DSHB, C. Goodman |
| mouse anti-Spacemaker | 1:50 | 21A6, DSHB, S. Benzer |
| mouse anti-Tubulin 85E | 1:3 | A. Salzberg (Klein et al., 2010) |
| mouse anti-Futsch | 1:25 | 22C10, DSHB, S. Benzer/N.Colley, |
| mouse anti-Eyes absent | 1:75 | Eya10H6, DSHB, S. Benzer/N.M.Bonini |
| guinea pig anti-Scute | 1:100 | S. Crews (Stagg et al., 2011) |
| mouse anti-GFP | 1:300 | ab1218, Abcam |
| rabbit anti-GFP | 1:1000 | 06-896 Milipore |
| mouse anti-GFP | 1:1000 | A-11120 Invitrogen |
| rabbit ant dsRED | 1:1000 | 632496 Clontech |
