# Supplementary material for: Evolutionary changes in transcription factor coding sequence quantitatively alter sensory organ development and function
Source: eLife. 2017 Apr 13;6:e26402. doi: 10.7554/eLife.26402 (PMC5432213; doi:10.7554/eLife.26402)
Supplement: Supplementary file 4. — DOI: http://dx.doi.org/10.7554/eLife.26402.020 [file elife-26402-supp4.docx]

**Supplementary File 4.** **Codon adaptation index (CAI) of the genes used in this study**

| **Gene** | **CAI**  Equation Sharp & Li | **CAI**  Equation Eyre-Walker |
| --- | --- | --- |
| Ato | 0.465 | 0.509 |
| Amos | 0.375 | 0.457 |
| BfAth | 0.308 | 0.397 |
| MmAth1 | 0.340 | 0.414 |
| MmAth5 | 0.388 | 0.534 |
| PdAth2 | 0.307 | 0.335 |
| Cato | 0.352 | 0.446 |
| HsAth1 | 0.318 | 0.409 |
| BmAto | 0.225 | 0.276 |
| Scute | 0.255 | 0.296 |
| Tap | 0.367 | 0.428 |
| BmAto_optimized | 0.715 | 0.812 |
| PdAth2_optimized | 0.737 | 0.789 |
