# Supplementary material for: Evolutionary changes in transcription factor coding sequence quantitatively alter sensory organ development and function
Source: eLife. 2017 Apr 13;6:e26402. doi: 10.7554/eLife.26402 (PMC5432213; doi:10.7554/eLife.26402)
Supplement: Supplementary file 5. — DOI: http://dx.doi.org/10.7554/eLife.26402.021 [file elife-26402-supp5.docx]

**Supplementary File 5.** **Primer pairs used for qPCR**

| **Gene** | **Primer1** | **Primer2** |
| --- | --- | --- |
| RPS13 | AGGGTCTGACTCCCTCCAAAA | AACGCACCTGGGCAACTC |
| Ato | CGATGGCAATGATGGATCCT | CCGGCAGCGGCATCT |
| Amos | ATGTATTACGATACGCCGTC | GAGGCGGAGGATGTAGA |
| BfAth | CCAACATCCTCCGCTAC | CTGTGCCATCTGAAGTG |
| MmAth1 | GCTGTGCAAGCTGAAGGG | TCTTGTCGTTGTTGAAGG |
| MmAth5 | AGGACAAGAAGCTGTCCAA | CCCGGGAAAGGGAGGTA |
| PdAth2 | GCCTACGAGGACGCTAA | GTATGATTCGGGACTCTGTG |
| AqbHLH1 | AACCCACCTCTCCCTCA | AAGGACAGCTCTCGTAATC |
