# Supplementary material for: Evolutionary changes in transcription factor coding sequence quantitatively alter sensory organ development and function
Source: eLife. 2017 Apr 13;6:e26402. doi: 10.7554/eLife.26402 (PMC5432213; doi:10.7554/eLife.26402)
Supplement: Supplementary file 6. — (A) Summary statistics of Figure 2D; number of observations (N), mean, median, standard deviation, 95% confidence interval and p-value of Shapiro Wilk test. (B) p-values of Figure 2D; p-values (t-test) and adjusted p-values (by Holm method). (C) Summary statistics of Figure 3B; number of observations (N), mean, median, standard deviation, 95% confidence interval and p-value of Shapiro Wilk test. (D) p-values of Figure 3B; p-values (Fisher exact test) and adjusted p-values (by Holm method). (E) Summary statistics of Figure 5B for bristles; number of observations (N), mean, median, standard deviation, 95% confidence interval and p-value of Shapiro Wilk test. (F) Summary statistics of Figure 5B for campaniform organs; number of observations (N), mean, median, standard deviation, 95% confidence interval and p-value of Shapiro Wilk test. (G) p-values of Figure 5B for bristles; p-values (Wilcoxon Rank Sum and Signed Rank Tests) and adjusted p-values (by Holm method). (H) p-values of Figure 5B for campaniform organs; p-values (Wilcoxon Rank Sum and Signed Rank Tests) and adjusted p-values (by Holm method) (I) Summary statistics of Figure 5C for bristles; number of observations (N), mean, median, standard deviation, 95% confidence interval and p-value of Shapiro Wilk test. (J) Summary statistics of Figure 5C for campaniform organs; number of observations (N), mean, median, standard deviation, 95% confidence interval and p-value of Shapiro Wilk test. (K) p-values of Figure 5C for bristles; p-values (Wilcoxon Rank Sum and Signed Rank Tests) and adjusted p-values (by Holm method). (L) p-values of Figure 5C for campaniform organs; p-values (Wilcoxon Rank Sum and Signed Rank Tests) and adjusted p-values (by Holm method). (M) Summary statistics of Figure 5D for bristles; number of observations (N), mean, median, standard deviation, 95% confidence interval and p-value of Shapiro Wilk test. (N) Summary statistics of Figure 5D for campaniform organs; number of observations (N), mean [file elife-26402-supp6.docx]

**Supplementary File 6**
**(A) Summary statistics of Figure 2D**; number of observations (N), mean, median, standard deviation, 95% confidence interval and p-value of Shapiro Wilk test.

| Genotyps | N | Mean | Median | StandardDev | Confi95 | ShapiroTest |
| --- | --- | --- | --- | --- | --- | --- |
| WT | 17 | 22,58824 | 23 | 1,502449 | 0,772488 | 1,945E-01 |
| Ato KI | 19 | 22,68421 | 23 | 1,492672 | 0,719445 | 6,689E-02 |
| Amos KI | 16 | 22,8125 | 23 | 1,682013 | 0,896281 | 2,806E-01 |
| BfAth KI | 17 | 20,11765 | 20 | 1,49509 | 0,768704 | 8,944E-02 |
| MmAth5 KI | 14 | 17,28571 | 17,5 | 1,382783 | 0,798395 | 1,395E-01 |
| PdAth2 KI | 14 | 16,21429 | 16 | 1,847184 | 1,066532 | 2,494E-01 |
| AqbHLH1 KI | 17 | 20,11765 | 20 | 1,268974 | 0,652446 | 1,297E-01 |

**(B)** **p-values of Figure 2D**; p-values (t-test) and adjusted p-values (by Holm method)

| Genotype1 | Genotype2 | p-value | Adjusted p-value |
| --- | --- | --- | --- |
| WT | Ato KI | 0,8489551 | 1 |
| WT | Amos KI | 0,6897646 | 1 |
| WT | BfAth KI | 3,49E-05 | 0,000209 |
| WT | MmAth5 KI | 4,73E-11 | 6,62E-10 |
| WT | PdAth2 KI | 1,48E-10 | 1,48E-09 |
| Ato KI | Amos KI | 0,8145827 | 1 |
| Ato KI | BfAth KI | 1,15E-05 | 8,05E-05 |
| Ato KI | MmAth5 KI | 1,19E-11 | 1,78E-10 |
| Ato KI | PdAth2 KI | 9,22E-11 | 1,20E-09 |
| Amos KI | BfAth KI | 3,51E-05 | 0,000209 |
| Amos KI | MmAth5 KI | 1,32E-10 | 1,45E-09 |
| Amos KI | PdAth2 KI | 1,16E-10 | 1,39E-09 |
| BfAth KI | MmAth5 KI | 7,22E-06 | 5,77E-05 |
| BfAth KI | PdAth2 KI | 1,15E-06 | 1,04E-05 |
| MmAth5 KI | PdAth2 KI | 0,0950875 | 0,38035 |

**(C)** **Summary statistics of Figure 3B**; number of observations (N), mean, median, standard deviation, 95% confidence interval and p-value of Shapiro Wilk test.

| Genotype | N | Mean | Median | StandardDev | Confi95 | ShapiroTest |
| --- | --- | --- | --- | --- | --- | --- |
| WT | 51 | 5,02E+00 | 5,00E+00 | 0,244147 | 0,0686675 | 4,23E-14 |
| Ato KI | 52 | 4,90E+00 | 5,00E+00 | 0,602596 | 0,167763851 | 1,13E-14 |
| Amos KI | 56 | 4,96E+00 | 5,00E+00 | 0,328317 | 0,08792393 | 2,88E-13 |
| BfAth KI | 61 | 4,64E+00 | 5,00E+00 | 0,65911 | 0,168805955 | 1,40E-12 |
| MmAth1 KI | 45 | 4,96E+00 | 5,00E+00 | 0,424026 | 0,127391489 | 7,76E-12 |
| MmAth5 KI | 46 | 2,76E+00 | 3,00E+00 | 0,99297 | 0,294875828 | 0,000177 |
| PdAth2 KI | 54 | 2,89E+00 | 3,00E+00 | 1,021775 | 0,278891023 | 0,000375 |
| AqbHLH1 KI | 48 | 3,29E+00 | 3,00E+00 | 0,849489 | 0,246665832 | 1,11E-06 |

**(D)** **p-values of Figure 3B**; p-values (Fisher exact test) and adjusted p-values (by Holm method)

| Genotype1 | Genotype2 | p-value | Adjusted p-valueP |
| --- | --- | --- | --- |
| WT | Ato KI | 1 | 1 |
| WT | Amos KI | 0,50792 | 1 |
| WT | BfAth KI | 3,91E-05 | 0,000509 |
| WT | MmAth5 KI | 5,25E-23 | 1,16E-21 |
| WT | PdAth2 KI | 1,42E-25 | 3,82E-24 |
| WT | AqbHLH1 KI | 1,04E-25 | 2,92E-24 |
| WT | MmAth1 KI | 7,75E-01 | 1,00E+00 |
| Ato KI | Amos KI | 7,08E-01 | 1,00E+00 |
| Ato KI | BfAth KI | 3,87E-04 | 4,65E-03 |
| Ato KI | MmAth5 KI | 7,24E-22 | 1,45E-20 |
| Ato KI | PdAth2 KI | 4,43E-24 | 1,11E-22 |
| Ato KI | AqbHLH1 KI | 5,66E-24 | 1,36E-22 |
| Ato KI | MmAth1 KI | 8,41E-01 | 1,00E+00 |
| Amos KI | BfAth KI | 1,41E-03 | 1,27E-02 |
| Amos KI | MmAth5 KI | 1,27E-22 | 2,67E-21 |

**(E)** **Summary statistics of Figure 5B for bristles**; number of observations (N), mean, median, standard deviation, 95% confidence interval and p-value of Shapiro Wilk test.

| Genotype | N | MeanBristles | MedianBristles | StandardDevBristles | Confi95Bristles | ShapiroTestBristles |
| --- | --- | --- | --- | --- | --- | --- |
| WT | 14 | 0 | 0 | 0 | 0 | NA |
| UAS Ato | 14 | 25,642857 | 26 | 2,762584 | 1,595068 | 2,424E-01 |
| UAS Amos | 16 | 20,3125 | 20,5 | 3,300884 | 1,758917 | 4,236E-02 |
| UAS Cato | 12 | 5,50E+00 | 4,5 | 2,354879 | 1,496219 | 1,339E-02 |
| UAS BfAth | 12 | 1,51E+01 | 1,60E+01 | 3,396745 | 2,158189 | 3,212E-01 |
| UAS BmAto | 10 | 9,00E-01 | 1,00E+00 | 0,994429 | 0,711372 | 3,252E-02 |
| UAS MmAth1 | 10 | 6,50E+01 | 6,40E+01 | 4,447221 | 3,181351 | 8,298E-01 |
| UAS MmAth5 | 16 | 0 | 0 | 0 | 0 | NA |
| UAS HsAth1 | 14 | 6,86E+01 | 6,75E+01 | 9,278653 | 5,357335 | 8,131E-01 |
| UAS PdAth2 | 16 | 1,06E+00 | 1,00E+00 | 0,680074 | 0,362386 | 1,085E-04 |
| UAS AqbHLH1 | 16 | 3,50E+00 | 3,00E+00 | 1,75119 | 0,933143 | 1,102E-01 |

**(F)** **Summary statistics of Figure 5B for campaniform organs**; number of observations (N), mean, median, standard deviation, 95% confidence interval and p-value of Shapiro Wilk test.

| Genotype | N | MeanCamp | MedianCamp | StandardDevCamp | Confi95Camp | ShapiroTestCamp |
| --- | --- | --- | --- | --- | --- | --- |
| WT | 14 | 4,142857 | 4 | 0,363137 | 0,209669 | 1,71E-06 |
| UAS Ato | 14 | 9,142857 | 8,5 | 2,684919 | 1,550226 | 0,136351 |
| UAS Amos | 16 | 11,875 | 12 | 2,418677 | 1,288822 | 0,454659 |
| UAS Cato | 12 | 6,25 | 6 | 1,05529 | 0,670499 | 0,068901 |
| UAS BfAth | 12 | 7,5 | 7,5 | 2,067058 | 1,313346 | 0,291791 |
| UAS BmAto | 10 | 5,5 | 6 | 0,849837 | 0,607936 | 0,000118 |
| UAS MmAth1 | 10 | 21 | 21,5 | 4,898979 | 3,504519 | 0,087436 |
| UAS HsAth1 | 14 | 10,85714 | 10,5 | 3,43863 | 1,985406 | 0,182553 |
| UAS MmAth5 | 16 | 4,125 | 4 | 0,341565 | 0,182007 | 3,41E-07 |
| UAS PdAth2 | 16 | 4,6875 | 4 | 1,078193 | 0,574528 | 0,020502 |
| UAS AqbHLH1 | 16 | 4,1875 | 4 | 1,424488 | 0,759056 | 0,053946 |

**(G) p-values of Figure 5B for bristles;** p-values (Wilcoxon Rank Sum and Signed Rank Tests) and adjusted p-values (by Holm method)

| Genotype1 | Genotype2 | p-value | Adjusted p-value |
| --- | --- | --- | --- |
| WT | UAS Ato | 1,64E-06 | 6,01E-05 |
| WT | UAS Amos | 9,64E-07 | 3,66E-05 |
| WT | UAS Cato | 2,65E-06 | 8,20E-05 |
| WT | UAS BfAth | 2,89E-06 | 8,67E-05 |
| WT | UAS BmAto | 0,001344 | 0,004032 |
| WT | UAS MmAth1 | 5,50E-06 | 0,000137 |
| WT | UAS HsAth1 | 1,68E-06 | 6,01E-05 |
| WT | UAS MmAth5 | NA | NA |
| WT | UAS PdAth2 | 4,78E-06 | 0,000124 |
| UAS Ato | UAS Amos | 0,000156 | 0,001093 |
| UAS Ato | UAS Cato | 1,57E-05 | 0,00033 |
| UAS Ato | UAS BfAth | 1,67E-05 | 0,00033 |
| UAS Ato | UAS BmAto | 4,23E-05 | 0,000635 |
| UAS Ato | UAS MmAth1 | 4,55E-05 | 0,000635 |
| UAS Ato | UAS HsAth1 | 7,28E-06 | 0,000175 |
| UAS Ato | UAS MmAth5 | 4,74E-07 | 2,04E-05 |
| UAS Ato | UAS PdAth2 | 1,63E-06 | 6,01E-05 |
| UAS Amos | UAS Cato | 8,27E-06 | 0,00019 |
| UAS Amos | UAS BfAth | 0,000794 | 0,003359 |
| UAS Amos | UAS BmAto | 2,49E-05 | 0,000423 |
| UAS Amos | UAS MmAth1 | 2,64E-05 | 0,000423 |
| UAS Amos | UAS HsAth1 | 3,42E-06 | 9,91E-05 |
| UAS Amos | UAS MmAth5 | 2,68E-07 | 1,18E-05 |
| UAS Amos | UAS PdAth2 | 7,63E-07 | 3,13E-05 |
| UAS Cato | UAS BfAth | 6,89E-05 | 0,000827 |
| UAS Cato | UAS BmAto | 8,12E-05 | 0,000855 |
| UAS Cato | UAS MmAth1 | 7,77E-05 | 0,000855 |
| UAS Cato | UAS HsAth1 | 1,61E-05 | 0,00033 |
| UAS Cato | UAS MmAth5 | 8,05E-07 | 3,22E-05 |
| UAS Cato | UAS PdAth2 | 4,00E-06 | 0,000108 |
| UAS BfAth | UAS BmAto | 7,77E-05 | 0,000855 |
| UAS BfAth | UAS MmAth1 | 8,50E-05 | 0,000855 |
| UAS BfAth | UAS HsAth1 | 1,71E-05 | 0,00033 |
| UAS BfAth | UAS MmAth5 | 8,73E-07 | 3,40E-05 |
| UAS BfAth | UAS PdAth2 | 3,80E-06 | 0,000106 |
| UAS BmAto | UAS MmAth1 | 0,000162 | 0,001093 |
| UAS BmAto | UAS HsAth1 | 4,35E-05 | 0,000635 |
| UAS BmAto | UAS MmAth5 | 0,000672 | 0,003359 |
| UAS BmAto | UAS PdAth2 | 0,412827 | 0,608484 |
| UAS MmAth1 | UAS HsAth1 | 0,304242 | 0,608484 |
| UAS MmAth1 | UAS MmAth5 | 1,72E-06 | 6,01E-05 |
| UAS MmAth1 | UAS PdAth2 | 1,02E-05 | 0,000224 |
| UAS HsAth1 | UAS MmAth5 | 4,86E-07 | 2,04E-05 |
| UAS HsAth1 | UAS PdAth2 | 1,66E-06 | 6,01E-05 |
| UAS MmAth5 | UAS PdAth2 | 1,68E-06 | 6,01E-05 |

**(H) p-values of Figure 5B for campaniform organs;** p-values (Wilcoxon Rank Sum and Signed Rank Tests) and adjusted p-values (by Holm method)

| Genotype1 | Genotype2 | p-value | Adjusted p-value |
| --- | --- | --- | --- |
| WT | UAS Ato | 2,94E-06 | 1,17E-04 |
| WT | UAS Amos | 1,59E-06 | 6,51E-05 |
| WT | UAS Cato | 1,11E-05 | 3,67E-04 |
| WT | UAS BfAth | 3,95E-05 | 1,11E-03 |
| WT | UAS BmAto | 0,000484 | 0,007259 |
| WT | UAS MmAth1 | 1,35E-05 | 0,000431 |
| WT | UAS HsAth1 | 2,96E-06 | 1,17E-04 |
| WT | UAS MmAth5 | 0,915704 | 0,915704 |
| WT | UAS PdAth2 | 1,29E-01 | 0,775966 |
| UAS Ato | UAS Amos | 0,010083 | 0,110918 |
| UAS Ato | UAS Cato | 2,62E-03 | 0,034033 |
| UAS Ato | UAS BfAth | 2,01E-01 | 0,792186 |
| UAS Ato | UAS BmAto | 1,64E-04 | 0,003259 |
| UAS Ato | UAS MmAth1 | 4,47E-05 | 0,001206 |
| UAS Ato | UAS HsAth1 | 1,93E-01 | 0,792186 |
| UAS Ato | UAS MmAth5 | 9,73E-07 | 4,28E-05 |
| UAS Ato | UAS PdAth2 | 1,04E-05 | 3,55E-04 |
| UAS Amos | UAS Cato | 1,01E-05 | 0,000355 |
| UAS Amos | UAS BfAth | 0,000117 | 0,002564 |
| UAS Amos | UAS BmAto | 2,20E-05 | 0,000639 |
| UAS Amos | UAS MmAth1 | 5,85E-05 | 0,001522 |
| UAS Amos | UAS HsAth1 | 4,15E-01 | 8,30E-01 |
| UAS Amos | UAS MmAth5 | 4,96E-07 | 2,23E-05 |
| UAS Amos | UAS PdAth2 | 1,21E-06 | 5,09E-05 |
| UAS Cato | UAS BfAth | 8,15E-02 | 0,652267 |
| UAS Cato | UAS BmAto | 1,58E-01 | 0,792186 |
| UAS Cato | UAS MmAth1 | 7,66E-05 | 0,001916 |
| UAS Cato | UAS HsAth1 | 4,04E-04 | 0,006458 |
| UAS Cato | UAS MmAth5 | 3,84E-06 | 1,46E-04 |
| UAS Cato | UAS PdAth2 | 1,74E-03 | 0,024374 |
| UAS BfAth | UAS BmAto | 7,26E-03 | 0,087148 |
| UAS BfAth | UAS MmAth1 | 8,07E-05 | 0,001916 |
| UAS BfAth | UAS HsAth1 | 2,08E-02 | 0,208294 |
| UAS BfAth | UAS MmAth5 | 1,51E-05 | 4,69E-04 |
| UAS BfAth | UAS PdAth2 | 3,69E-04 | 0,006278 |
| UAS BmAto | UAS MmAth1 | 0,000129 | 0,002716 |
| UAS BmAto | UAS HsAth1 | 7,92E-05 | 0,001916 |
| UAS BmAto | UAS MmAth5 | 0,000225 | 0,004048 |
| UAS BmAto | UAS PdAth2 | 0,053089 | 0,477801 |
| UAS MmAth1 | UAS HsAth1 | 0,000163 | 0,003259 |
| UAS MmAth1 | UAS MmAth5 | 5,06E-06 | 1,87E-04 |
| UAS MmAth1 | UAS PdAth2 | 2,05E-05 | 0,000616 |
| UAS HsAth1 | UAS MmAth5 | 9,79E-07 | 4,28E-05 |
| UAS HsAth1 | UAS PdAth2 | 5,99E-06 | 2,16E-04 |
| UAS MmAth5 | UAS PdAth2 | 9,31E-02 | 6,52E-01 |

**(I)** **Summary statistics of Figure 5C for bristles**; number of observations (N), mean, median, standard deviation, 95% confidence interval and p-value of Shapiro Wilk test.

| Genotype | N | MeanBristles | MedianBristles | StandardDevBristles | Confi95Bristles | ShapiroTestBristles |
| --- | --- | --- | --- | --- | --- | --- |
| WT | 16 | 0,00E+00 | 0,00E+00 | 0 | 0 | NA |
| UAS Ato | 16 | 2,89E+01 | 2,90E+01 | 5,690636 | 3,032326 | 3,04E-01 |
| UAS BmAto | 16 | 1,81E+00 | 2,00E+00 | 1,167262 | 0,62199 | 0,213242 |
| UAS BmAto opti | 16 | 5,06E+00 | 5,00E+00 | 2,143789 | 1,142345 | 0,054856 |
| UAS PdAth2 | 16 | 9,38E-01 | 5,00E-01 | 1,12361 | 0,59873 | 0,001946 |
| UAS PdAth2 opti | 16 | 1,6875 | 1 | 1,078193 | 0,574528 | 0,020502 |

**(J)** **Summary statistics of Figure 5C for campaniform organs**; number of observations (N), mean, median, standard deviation, 95% confidence interval and p-value of Shapiro Wilk test.

| Genotype | N | MeanCamp | MedianCamp | StandardDevCamp | Confi95Camp | ShapiroTestCamp |
| --- | --- | --- | --- | --- | --- | --- |
| WT | 16 | 4,25E+00 | 4,00E+00 | 0,447214 | 0,238303 | 5,27E-06 |
| UAS Ato | 16 | 9,19E+00 | 9,00E+00 | 2,166987 | 1,154706 | 0,955181 |
| UAS BmAto | 16 | 5,13E+00 | 5,00E+00 | 1,454877 | 0,775249 | 0,04762 |
| UAS BmAto opti | 16 | 6,06E+00 | 6,00E+00 | 1,436141 | 0,765265 | 0,088281 |
| UAS PdAth2 | 16 | 4,6875 | 5 | 1,138347 | 0,606582 | 0,114017 |
| UAS PdAth2 opti | 16 | 4,75E+00 | 5 | 1 | 0,532862 | 0,094129 |

**(K)** **p-values of Figure 5C for bristles;** p-values (Wilcoxon Rank Sum and Signed Rank Tests) and adjusted p-values (by Holm method)

| Genotype1 | Genotype2 | p-value | Adjusted p-value |
| --- | --- | --- | --- |
| WT | UAS Ato | 2,77E-07 | 2,64E-06 |
| WT | UAS BmAto | 3,24E-06 | 1,62E-05 |
| WT | UAS BmAto opti | 2,64E-07 | 2,64E-06 |
| WT | UAS PdAth2 | 1,63E-03 | 3,25E-03 |
| UAS Ato | UAS BmAto | 1,40E-06 | 9,95E-06 |
| UAS Ato | UAS BmAto opti | 1,46E-06 | 9,95E-06 |
| UAS Ato | UAS PdAth2 | 1,24E-06 | 9,95E-06 |
| UAS BmAto | UAS BmAto opti | 8,48E-05 | 0,000254 |
| UAS BmAto | UAS PdAth2 | 3,91E-02 | 0,03913 |
| UAS BmAto opti | UAS PdAth2 | 7,19E-06 | 2,87E-05 |

**(L)** **p-values of Figure 5C for campaniform organs;** p-values (Wilcoxon Rank Sum and Signed Rank Tests) and adjusted p-values (by Holm method)

| Genotype1 | Genotype2 | p-value | Adjusted p-value |
| --- | --- | --- | --- |
| WT | UAS Ato | 1,09E-06 | 1,09E-05 |
| WT | UAS BmAto | 3,29E-02 | 1,32E-01 |
| WT | UAS BmAto opti | 2,44E-04 | 1,47E-03 |
| WT | UAS PdAth2 | 1,48E-01 | 2,96E-01 |
| UAS Ato | UAS BmAto | 2,70E-05 | 0,000216 |
| UAS Ato | UAS BmAto opti | 1,96E-04 | 0,001374 |
| UAS Ato | UAS PdAth2 | 6,43E-06 | 5,78E-05 |
| UAS BmAto | UAS BmAto opti | 0,064614 | 0,193842 |
| UAS BmAto | UAS PdAth2 | 5,18E-01 | 0,518239 |
| UAS BmAto opti | UAS PdAth2 | 0,011513 | 0,057567 |

**(M)** **Summary statistics of Figure 5D for bristles**; number of observations (N), mean, median, standard deviation, 95% confidence interval and p-value of Shapiro Wilk test.

| Genotype | N | MeanBristles | MedianBristles | StandardDevBristles | Confi95Bristles | ShapiroTestBristles |
| --- | --- | --- | --- | --- | --- | --- |
| WT | 16 | 0,00E+00 | 0 | 0 | 0 | NA |
| UAS Ato | 16 | 2,44E+01 | 2,45E+01 | 4,440345 | 2,36609301 | 0,260064 |
| UAS Ato w bHLHMmAth1 | 16 | 0,00E+00 | 0 | 0 | 0 | NA |
| UAS Ato w bHLHPdAth2 | 14 | 9,50E+00 | 9,5 | 2,345208 | 1,354082716 | 0,278536 |
| UAS MmAth1 | 16 | 6,88E+01 | 67 | 7,995832 | 4,260678253 | 0,551936 |
| UAS MmAth1 w bHLHAto | 15 | 2,77E+01 | 28 | 4,4955 | 2,489525139 | 0,303294 |
| UAS PdAth2 | 29 | 1,86E+00 | 2 | 1,457104 | 0,554252803 | 0,00753 |
| UAS PdAth2 w bHLHAto | 16 | 1,50E+00 | 1,00E+00 | 1,264911 | 0,674023528 | 0,017014 |

**(N)** **Summary statistics of Figure 5D for campaniform organs**; number of observations (N), mean, median, standard deviation, 95% confidence interval and p-value of Shapiro Wilk test.

| Genotype | N | MeanCamp | MedianCamp | StandardDevCamp | Confi95Camp | ShapiroTestCamp |
| --- | --- | --- | --- | --- | --- | --- |
| WT | 16 | 4,00E+00 | 4,00E+00 | 0 | 0 | NA |
| UAS Ato | 16 | 7,88E+00 | 8 | 1,78419 | 0,950728 | 0,178366 |
| UAS Ato w bHLHMmAth1 | 16 | 4,13E+00 | 4 | 0,341565 | 0,182007 | 3,41E-07 |
| UAS Ato w bHLHPdAth2 | 14 | 7,14E+00 | 7 | 1,167321 | 0,673991 | 0,370275 |
| UAS MmAth1 | 16 | 1,54E+01 | 15 | 3,650913 | 1,945434 | 0,714997 |
| UAS MmAth1 w bHLHAto | 15 | 1,07E+01 | 1,00E+01 | 2,742956 | 1,518999 | 0,327889 |
| UAS PdAth2 | 29 | 4,83E+00 | 5,00E+00 | 0,966177 | 0,367514 | 0,000561 |
| UAS PdAth2 w bHLHAto | 16 | 4,00E+00 | 4 | 1,21106 | 0,645328 | 0,233722 |

**(O)** **p-values of Figure 5D for bristles;** p-values (Wilcoxon Rank Sum and Signed Rank Tests) and adjusted p-values (by Holm method)

| Genotype1 | Genotype2 | p-value | Adjusted p-value |
| --- | --- | --- | --- |
| WT | UAS Ato | 2,70E-07 | 4,33E-06 |
| WT | UAS Ato w bHLHMmAth1 | NA | NA |
| WT | UAS Ato w bHLHPdAth2 | 4,56E-07 | 4,56E-06 |
| WT | UAS MmAth1 | 2,77E-07 | 4,33E-06 |
| WT | UAS MmAth1 w bHLHAto | 3,60E-07 | 4,33E-06 |
| WT | UAS PdAth2 | 1,40E-06 | 1,12E-05 |
| UAS Ato | UAS Ato w bHLHMmAth1 | 2,70E-07 | 4,33E-06 |
| UAS Ato | UAS Ato w bHLHPdAth2 | 3,29E-06 | 1,32E-05 |
| UAS Ato | UAS MmAth1 | 1,48E-06 | 1,12E-05 |
| UAS Ato | UAS MmAth1 w bHLHAto | 7,09E-02 | 7,09E-02 |
| UAS Ato | UAS PdAth2 | 3,18E-08 | 6,36E-07 |
| UAS Ato w bHLHMmAth1 | UAS Ato w bHLHPdAth2 | 4,56E-07 | 4,56E-06 |
| UAS Ato w bHLHMmAth1 | UAS MmAth1 | 2,77E-07 | 4,33E-06 |
| UAS Ato w bHLHMmAth1 | UAS MmAth1 w bHLHAto | 3,60E-07 | 4,33E-06 |
| UAS Ato w bHLHMmAth1 | UAS PdAth2 | 1,40E-06 | 1,12E-05 |
| UAS Ato w bHLHPdAth2 | UAS MmAth1 | 3,36E-06 | 1,32E-05 |
| UAS Ato w bHLHPdAth2 | UAS MmAth1 w bHLHAto | 4,75E-06 | 1,32E-05 |
| UAS Ato w bHLHPdAth2 | UAS PdAth2 | 1,92E-07 | 3,26E-06 |
| UAS MmAth1 | UAS MmAth1 w bHLHAto | 2,25E-06 | 1,13E-05 |
| UAS MmAth1 | UAS PdAth2 | 3,21E-08 | 6,36E-07 |
| UAS MmAth1 w bHLHAto | UAS PdAth2 | 6,10E-08 | 1,10E-06 |

**(P) p-values of Figure 5D for campaniform organs;** p-values (Wilcoxon Rank Sum and Signed Rank Tests) and adjusted p-values (by Holm method)

| Genotype1 | Genotype2 | p-value | Adjusted p-value |
| --- | --- | --- | --- |
| WT | UAS Ato | 2,56E-07 | 4,86E-06 |
| WT | UAS Ato w bHLHMmAth1 | 1,64E-01 | 3,27E-01 |
| WT | UAS Ato w bHLHPdAth2 | 4,41E-07 | 7,05E-06 |
| WT | UAS MmAth1 | 2,75E-07 | 4,95E-06 |
| WT | UAS MmAth1 w bHLHAto | 3,49E-07 | 5,94E-06 |
| WT | UAS PdAth2 | 2,82E-04 | 1,97E-03 |
| UAS Ato | UAS Ato w bHLHMmAth1 | 5,80E-07 | 7,54E-06 |
| UAS Ato | UAS Ato w bHLHPdAth2 | 2,76E-01 | 3,27E-01 |
| UAS Ato | UAS MmAth1 | 4,26E-06 | 3,56E-05 |
| UAS Ato | UAS MmAth1 w bHLHAto | 4,30E-03 | 1,29E-02 |
| UAS Ato | UAS PdAth2 | 4,71E-07 | 7,07E-06 |
| UAS Ato w bHLHMmAth1 | UAS Ato w bHLHPdAth2 | 1,14E-06 | 1,26E-05 |
| UAS Ato w bHLHMmAth1 | UAS MmAth1 | 5,08E-07 | 7,11E-06 |
| UAS Ato w bHLHMmAth1 | UAS MmAth1 w bHLHAto | 6,84E-07 | 8,21E-06 |
| UAS Ato w bHLHMmAth1 | UAS PdAth2 | 3,05E-03 | 1,22E-02 |
| UAS Ato w bHLHPdAth2 | UAS MmAth1 | 3,96E-06 | 3,56E-05 |
| UAS Ato w bHLHPdAth2 | UAS MmAth1 w bHLHAto | 3,40E-04 | 2,04E-03 |
| UAS Ato w bHLHPdAth2 | UAS PdAth2 | 2,94E-06 | 2,94E-05 |
| UAS MmAth1 | UAS MmAth1 w bHLHAto | 9,79E-04 | 4,89E-03 |
| UAS MmAth1 | UAS PdAth2 | 2,26E-08 | 4,75E-07 |
| UAS MmAth1 w bHLHAto | UAS PdAth2 | 6,47E-08 | 1,29E-06 |

**(Q)** **Summary statistics for passive antennal mechanics (best frequency, f0; tuning sharpness, Q; apparent mass) across ATH rescues (relating to Figure 4A)**; number of observations (N), mean, median, standard deviation, standard error, 95% confidence interval and p-values for pair-wise comparison with Ato KI control (t-test for normally distributed data and Mann-Whitney Rank Sum test (MWRS) for non-normally distributed data. Significances are highlighted in color (<0.05 and <0.001).

| Genotype | Parameter | N | Mean | Median | StandardDev | StandardErr | 95% | p-value |
| --- | --- | --- | --- | --- | --- | --- | --- | --- |
| Ato KI | f0 (Hz) | 7 | 750.23 | 743.30 | 49.24 | 18.61 | 45.54 | -- |
|  | Q |  | 1.05 | 1.01 | 0.20 | 0.08 | 0.19 | -- |
|  | mass (kg) |  | 5.33*10^-12^ | 4.78*10^-12^ | 1.71*10^-12^ | 0.64*10^-12^ | 1.58*10^-12^ | -- |
| Amos KI | f0 (Hz) | 7 | 714.07 | 698.09 | 43.20 | 16.33 | 39.96 | 0.170 (t-test) |
|  | Q |  | 0.95 | 0.96 | 0.10 | 0.04 | 0.09 | 0.259 (t-test) |
|  | mass (kg) |  | 4.94*10^-12^ | 4.94*10^-12^ | 0.83*10^-12^ | 0.31*10^-12^ | 0.77*10^-12^ | 0.602 (t-test) |
| BfAth KI | f0 (Hz) | 7 | 720.99 | 713.71 | 33.83 | 13.81 | 35.50 | 0.246 (t-test) |
|  | Q |  | 1.14 | 1.14 | 0.04 | 0.02 | 0.04 | 0.101 (MWRS) |
|  | mass (kg) |  | 4.57*10^-12^ | 4.80*10^-12^ | 0.89*10^-12^ | 0.36*10^-12^ | 0.93*10^-12^ | 0.353 (t-test) |
| MmAth1 KI | f0 (Hz) | 6 | 716.11 | 716.37 | 67.79 | 27.68 | 71.14 | 0.316 (t-test) |
|  | Q |  | 0.74 | 0.73 | 0.06 | 0.03 | 0.07 | 0.00412 (t-test) |
|  | mass (kg) |  | 5.63*10^-12^ | 5.05*10^-12^ | 1.99*10^-12^ | 0.81*10^-12^ | 2.09*10^-12^ | 0.774 (t-tes) |
| MmAth5 KI | f0 (Hz) | 7 | 706.02 | 672.27 | 78.95 | 32.23 | 82.85 | 0.243 (t-test) |
|  | Q |  | 1.34 | 1.17 | 0.31 | 0.13 | 0.32 | 0.0720 (t-test) |
|  | mass (kg) |  | 5.12*10^-12^ | 4.60*10^-12^ | 1.79*10^-12^ | 0.73*10^-12^ | 1.88*10^-12^ | 0.945 (MWRS) |
| PdAth2 KI | f0 (Hz) | 11 | 2917.51 | 2915.67 | 1066.95 | 321.70 | 716.80 | <0.001 (MWRS) |
|  | Q |  | 1.64 | 1.62 | 0.57 | 0.17 | 0.38 | 0.019 (MWRS) |
|  | mass (kg) |  | 2.30*10^-12^ | 2.21*10^-12^ | 0.70*10^-12^ | 0.21*10^-12^ | 0.47*10^-12^ | <0.001 (t-test) |
| AqbHLH1 KI | f0 (Hz) | 6 | 726.64 | 705.70 | 103.57 | 42.28 | 108.69 | 0.601 (t-test) |
|  | Q |  | 1.11 | 1.16 | 0.15 | 0.06 | 0.15 | 0.571 (t-test) |
|  | mass (kg) |  | 4.91*10^-12^ | 4.25*10^-12^ | 2.35*10^-12^ | 0.96*10^-12^ | 2.47*10^-12^ | 0.445 (MWRS) |

**(R)** **Summary statistics for active antennal mechanics (best frequency, f0; tuning sharpness, Q; energy gain) across ATH rescues (relating to Figure 4A)**; number of observations (N), mean, median, standard deviation, standard error, 95% confidence interval and p-values for pair-wise comparison with Ato KI control (t-test for normally distributed data and Mann-Whitney Rank Sum test (MWRS) for non-normally distributed data. Significances are highlighted in color (<0.05 and <0.001).

| Genotype | Parameter | N | Mean | Median | StandardDev | StandardErr | 95% | p-value |
| --- | --- | --- | --- | --- | --- | --- | --- | --- |
| Ato KI | f0 (Hz) | 7 | 266.50 | 250.68 | 96.26 | 20.52 | 42.68 | -- |
|  | Q |  | 1.23 | 1.16 | 0.44 | 0.10 | 0.21 | -- |
|  | Energy (kBT) |  | 6.47 | 5.22 | 2.97 | 1.12 | 2.75 | -- |
| Amos KI | f0 (Hz) | 7 | 285.01 | 298.99 | 56.57 | 12.65 | 26.48 | 0.141 (MWRS) |
|  | Q |  | 1.10 | 1.06 | 0.33 | 0.07 | 0.15 | 0.332 (MWRS) |
|  | Energy (kBT) |  | 6.75 | 6.47 | 3.74 | 0.72 | 1.48 | 0.517 (MWRS) |
| BfAth KI | f0 (Hz) | 7 | 330.08 | 286.68 | 138.80 | 32.72 | 69.02 | 0.080 (MWRS) |
|  | Q |  | 1.65 | 1.26 | 1.15 | 0.28 | 0.59 | 0.326 (MWRS) |
|  | Energy (kBT) |  | 6.80 | 6.44 | 2.83 | 0.65 | 1.37 | 0.795 (t-test) |
| MmAth1 KI | f0 (Hz) | 6 | 534.19 | 524.68 | 108.11 | 38.22 | 90.38 | <0.001 (MWRS) |
|  | Q |  | 4.53 | 4.21 | 1.51 | 0.53 | 1.26 | <0.001 (MWRS) |
|  | Energy (kBT) |  | 7.45 | 7.87 | 2.25 | 0.80 | 1.88 | 0.479 (t-test) |
| MmAth5 KI | f0 (Hz) | 7 | 565.18 | 544.82 | 87.47 | 20.07 | 42.16 | <0.001 (MWRS) |
|  | Q |  | 2.22 | 1.92 | 0.88 | 0.20 | 0.42 | <0.001 (MWRS) |
|  | Energy (kBT) |  | 4.15 | 4.08 | 1.89 | 0.38 | 0.78 | 0.0174 (t-test) |
| PdAth2 KI | f0 (Hz) | 11 | 2917.51 | 2915.67 | 1066.95 | 321.70 | 716.80 | <0.001 (MWRS) |
|  | Q |  | 1.64 | 1.62 | 0.57 | 0.17 | 0.38 | 0.007 (MWRS) |
|  | Energy (kBT) |  | 0.49 | 0.46 | 0.63 | 0.19 | 0.42 | <0.001 (MWRS) |
| AqbHLH1 KI | f0 (Hz) | 6 | 498.49 | 474.57 | 129.67 | 32.42 | 69.10 | <0.001 (MWRS) |
|  | Q |  | 1.52 | 1.49 | 0.42 | 0.11 | 0.23 | 0.028 (MWRS) |
|  | Energy (kBT) |  | 3.89 | 3.98 | 1.96 | 0.42 | 0.87 | 0.0127 (t-test) |

**(S)** **Summary statistics for gating compliance analysis (relating to Figure 4-figure supplement 1B) across ATH rescues (number of sensitive ion channels, Ns; number of insensitive ion channels, Ni; single channel gating force of sensitive ion channels, zs; single channel gating force of insensitive ion channels, zi; asymptotic stiffness, Kinf; steady-state stiffness, Ksteady; total gating spring stiffness, KGS)**; number of observations (N), mean, median, standard deviation, standard error, 95% confidence interval and p-values for pair-wise comparison with Ato KI control (t-test for normally distributed data and Mann-Whitney Rank Sum test (MWRS) for non-normally distributed data. Significances are highlighted in color (<0.05 and <0.001). Note that parameter values for the PdAth2 KI were dispensed from statistical comparison to the control condition, as the transduction system in the antennae of PdAth2 KI flies did not comply to the two transducer population model (from Effertz et al. 2012), but rather conformed to a one transducer population model. It was thus not immediately evident how to compare the single transducer populations of PdAth2 KI flies to the two transducer (sensitive and insensitive, respectively) populations of control flies.

| Genotype | Parameter | N | Mean | Median | StandardDev | StandardErr | 95% | p-value |
| --- | --- | --- | --- | --- | --- | --- | --- | --- |
| Ato KI | Ns | 7 | 218.01 | 226.58 | 106.53 | 28.47 | 61.51 | -- |
|  | Ni |  | 18433.13 | 16621.48 | 9299.07 | 2485.28 | 5369.24 | -- |
|  | zs (fN) |  | 43.14 | 41.58 | 8.90 | 2.38 | 5.14 | -- |
|  | zi (fN) |  | 5.45 | 5.11 | 1.36 | 0.36 | 0.78 | -- |
|  | Kinf (μN/m) |  | 73.97 | 73.66 | 5.3 | 2.0 | 4.9 | -- |
|  | Ksteady (μN/m) |  | 46.64 | 46.08 | 2.68 | 1.01 | 2.48 | -- |
|  | KGS (μN/m) |  | 24.07 | 23.62 | 4.09 | 1.09 | 2.36 | -- |
| Amos KI | Ns | 7 | 168.29 | 175.27 | 55.52 | 22.67 | 58.27 | 0.298 (t-test) |
|  | Ni |  | 19020.71 | 16382.83 | 6430.34 | 2625.18 | 6748.31 | 0.890 (t-test) |
|  | zs (fN) |  | 47.63 | 49.04 | 6.51 | 2.66 | 6.83 | 0.283 (t-test) |
|  | zi (fN) |  | 5.05 | 5.06 | 0.76 | 0.31 | 0.79 | 0.509 (t-test) |
|  | Kinf (μN/m) |  | 72.23 | 70.90 | 9.00 | 3.68 | 9.45 | 0.645 (t-test) |
|  | Ksteady (μN/m) |  | 46.73 | 45.38 | 6.18 | 2.52 | 6.48 | 0.562 (t-test) |
|  | KGS (μN/m) |  | 25.50 | 24.93 | 3.65 | 1.49 | 3.83 | 0.472 (t-test) |
| BfAth KI | Ns | 8 | 140.72 | 126.99 | 97.55 | 34.49 | 81.56 | 0.107 (t-tes) |
|  | Ni |  | 14406.86 | 12828.91 | 7325.98 | 2590.12 | 6124.81 | 0.307 (t-test) |
|  | zs (fN) |  | 55.17 | 53.98 | 15.11 | 5.34 | 12.63 | 0.044 (MWRS) |
|  | zi (fN) |  | 5.76 | 5.59 | 1.49 | 5.27 | 1.25 | 0.627 (t-test) |
|  | Kinf (μN/m) |  | 68.90 | 67.70 | 3.78 | 1.34 | 3.16 | 0.562 (MWRS) |
|  | Ksteady (μN/m) |  | 42.93 | 42.98 | 1.75 | 0.62 | 1.46 | 0.811 (MWRS) |
|  | KGS (μN/m) |  | 25.97 | 25.76 | 2.79 | 0.98 | 2.33 | 0.259 (t-test) |
| MmAth1 KI | Ns | 5 | 307.41 | 290.52 | 80.26 | 40.13 | 127.68 | 0.142 (t-test) |
|  | Ni |  | 53895.58 | 47761.53 | 19279.94 | 9639.97 | 30671.90 | <0.001 (t-test) |
|  | zs (fN) |  | 47.40 | 48.63 | 5.01 | 2.51 | 7.97 | 0.379 (t-test) |
|  | zi (fN) |  | 1.89 | 2.17 | 1.54 | 0.77 | 2.45 | <0.001 (t-test) |
|  | Kinf (μN/m) |  | 105.98 | 99.78 | 23.27 | 11.63 | 37.01 | <0.001 (t-test) |
|  | Ksteady (μN/m) |  | 64.12 | 63.12 | 12.60 | 6.30 | 20.05 | <0.001 (t-test) |
|  | KGS (μN/m) |  | 41.85 | 36.66 | 11.32 | 5.66 | 18.00 | 0.003 (t-test) |
| MmAth5 KI | Ns | 8 | 154.08 | 141.90 | 79.97 | 28.27 | 66.86 | 0.157 (t-tes) |
|  | Ni |  | 14999.37 | 13602.31 | 5181.58 | 1831.96 | 4332.00 | 0.350 (t-test) |
|  | zs (fN) |  | 55.68 | 50.97 | 11.37 | 4.02 | 9.51 | 0.00937 (t-test) |
|  | zi (fN) |  | 3.73 | 3.87 | 0.71 | 0.25 | 0.59 | 0.00351 (t-test) |
|  | Kinf (μN/m) |  | 87.26 | 83.84 | 9.94 | 3.51 | 8.31 | <0.001 (MWRS) |
|  | Ksteady (μN/m) |  | 60.02 | 59.48 | 6.63 | 2.34 | 5.54 | <0.001 (t-test) |
|  | KGS (μN/m) |  | 27.06 | 26.55 | 4.00 | 1.41 | 3.34 | 0.112 (t-test) |
| PdAth2 KI | Ns/Ni | 11 | 290.49 | 221.57 | 109.62 | 62.04 | 122.29 | -- |
|  | zs/zi (fN) |  | 16.28 | 14.01 | 9.53 | 4.99 | 10.64 | -- |
|  | Kinf (μN/m) |  | 212.72 | 237.8 | 77.8 | 27.5 | 65.1 | <0.001 (MWRS) |
|  | Ksteady (μN/m) |  | 100.73 | 109.97 | 30.55 | 10.80 | 25.54 | <0.001 (MWRS) |
|  | KGS (μN/m) |  | 116.45 | 127.26 | 48.35 | 17.09 | 40.42 | <0.001 (t-test) |
| AqbHLH1 KI | Ns | 8 | 66.97 | 63.55 | 21.08 | 7.97 | 19.50 | 0.004 (MWRS) |
|  | Ni |  | 11792.18 | 11697.02 | 6272.26 | 2370.69 | 5800.99 | 0.106 (t-test) |
|  | zs (fN) |  | 70.98 | 70.99 | 9.00 | 3.40 | 8.32 | <0.001 (t-test) |
|  | zi (fN) |  | 6.19 | 6.09 | 1.33 | 0.50 | 1.23 | 0.251 (t-test) |
|  | Kinf |  | 82.62 | 78.03 | 11.59 | 4.38 | 10.71 | 0.00215 (t-test) |
|  | Ksteady (μN/m) |  | 56.75 | 54.96 | 7.22 | 2.73 | 6.68 | <0.001 (t-test) |
|  | KGS (μN/m) |  | 25.87 | 25.17 | 5.24 | 1.98 | 4.84 | 0.397 (t-test) |
