# Supplementary material for: Evolutionary changes in transcription factor coding sequence quantitatively alter sensory organ development and function
Source: eLife. 2017 Apr 13;6:e26402. doi: 10.7554/eLife.26402 (PMC5432213; doi:10.7554/eLife.26402)
Supplement: Supplementary file 7. — DOI: http://dx.doi.org/10.7554/eLife.26402.023 [file elife-26402-supp7.docx]

### Supplementary File 7: Sequences codon optimized genes

>PdAth2_optimized
ATGTTCGATAGCAAGAGCCGCGATAAGATGCCCGATAACATGTTCAGCTGCATGGCCTACGAGGATGCCAAGGTGGGCATCCCCCTGCCCGCCACCATGACCCTGCCCCGCAGCAGCAGCCCCCTGTTCAGCGAGTTCAGCCCCGCCGATAGCAGCGTGAGCTTCGGCAGCAGCCTGGATAGCTGCTACGATCCCCAGAGCCCCGAGAGCTACCTGAACCTGCCCCCCTGCCAGCGCCAGAACAGCCCCGATAGCACCGGCACCGATCGCCAGGAGGATCTGGGCAGCAGCGATGATAGCGATCTGCAGGATAAGCCCACCAAGAAGGCCCGGGGGCGCAAGCGCGGCCCCTGCAAGGTGCCCGGCAAAGAGGTGGTGAAGCAGCGTCGGGTGGCTGCCAACGCCCGCGAACGACGTCGTATGCTGAGCCTGAACGTGGCCTTCGATAAGCTGCGCGATGTGGTGCCCGCCTTCAGCAGCGATCGCAAGCTGAGCAAGTACGAGACCCTGCAGATGGCCCAGAGCTACATCAGCGCCCTGCAGGAGCTGCTGACCAAGGATCCCGTGACCTAA

>BmAto_optimized
ATGACCGCCGAGACCTACGGCCACCGCCTGGTGTACAGCGAGAAGGATATCTTCAGCAACGATGTGATGCTGGAGTACGCCACCGAGGATTGCTACCTGACCTGGCCCCGCAGCCCCGATAGCGGCCGCAGCAGCCTGGAGCCCACCCCCAGCATCGATGGCAGCATCAGCCACGATAGCACCCACATCGCCTACCGCGGCCTGAGCCGCGATATGGTGCTGGAGGATAGCGCCGAGGATAACGATCTGCTGGAGGGCAGCGGCAAGAGGAGAGGGAGAGCAACCAGCGCAGCTGTCTTGAGGAGACGTCGGCTGGCCGCCAACGCCCGCGAGCGCCGCCGCATGCAGAACCTGAACAAGGCCTTCGATCGCCTGCGCGGCCACCTGCCCAGCCTGGGCGCCGATCGCCAGCTGAGCAAGTACGAGACCCTGCAGATGGCCCAGACCTACATCGCCGCCCTGTACGAGCTGCTGCAGTAA
